# Hydrothermal Synthesis of Cancrinite from Coal Gangue for the Immobilization of Sr

**DOI:** 10.3390/ma17030573

**Published:** 2024-01-25

**Authors:** Hao Wang, Fujie Zhang, Ran Ang, Ding Ren

**Affiliations:** Key Laboratory of Radiation Physics and Technology of Ministry of Education, Institute of Nuclear Science and Technology, Sichuan University, Chengdu 610065, China; wanghao720@stu.scu.edu.cn (H.W.); zhangfujie131@163.com (F.Z.); rang@scu.edu.cn (R.A.)

**Keywords:** coal gangue, cancrinite, hydrothermal synthesis, Sr, emergency curing

## Abstract

The primary objective of this study is to investigate and develop a rapid and effective method for the immobilization of Sr in the event of a nuclear leakage incident. Coal gangue, an underutilized form of solid waste from the coal industry, can be used as a raw material for curing Sr due to its high content of silica–alumina oxides. In the present study, Sr was successfully solidified in cancrinite synthesized using a hydrothermal method with coal gangue as raw material. A stable cancrinite phase was formed at a relative alkali concentration of more than 6 M. When the Sr/Al(Si) ratio was <1/6, cancrinite was the only stable phase that varied with the hydrothermal temperature and time. When the Sr/Al(Si) ratio increased to 1/2, the cancrinite phase completely disappeared, and a new strontium feldspar phase (SrAl_2_Si_2_O_8_) appeared. PCT leaching experiments showed that when Sr/Al(Si) < 1/6, the Sr leaching rate of Sr-cancrinite samples obtained by hydrothermal synthesis at 180 °C for 24 h was very low.

## 1. Introduction

Nuclear energy represents a highly concentrated and versatile energy source with extensive potential for application [1]. However, the development and utilization of nuclear technology, while yielding economic and societal benefits, also entail the inherent risk of nuclear accidents [2,3]. One such hazard involves the presence of ^90^Sr, a prominent constituent found in spent nuclear fuel, high-level liquid waste (HLLW) resulting from spent nuclear fuel reprocessing, and other waste materials generated during reactor operation [4,5]. In waste, ^90^Sr typically exists in ionic form and demonstrates strong migration capabilities in water [6]. In the event of a nuclear accident, the decay heat of radioactive strontium accelerates its diffusion and migration, posing severe consequences if the nuclide enters the biosphere [7]. During an emergency nuclear spill, the HLLW typically exhibits high mobility, making it challenging to collect and isolate effectively [8]. Consequently, a recommended approach involves the expedient solidification of the radionuclides present in the HLLW, evaluating their concentrations, and thereby diminishing its potential hazards for subsequent disposal purposes [9]. It is imperative for the development of nuclear energy to conduct applied basic research on the emergency treatment of radioactive strontium in leaking waste from nuclear accidents, as well as to develop rapid and efficient materials for nuclear emergency treatment.

The utilization of Synroc solidification techniques for certain radionuclides has demonstrated remarkable performance and holds significant prospects for further advancement [10,11,12,13,14,15]. The essence of its solidification treatment is to form a certain solid solution of the radionuclides in high-level liquid waste with the corresponding salts in the high-temperature phase, thereby encapsulating the radionuclides in the solid phase and creating a thermodynamically stable solidified matrix [16,17,18,19]. Researchers have employed natural zeolite as an adsorption material to effectively remove Sr^2+^ and Cs^+^ ions from aqueous solutions, yielding positive outcomes [20]. Cancrinite, an exceptional zeolite, exhibits an open framework structure comprising hexagonal rings of SiO_4_ and AlO_4_ tetrahedra stacked along the hexagonal c-axis [21,22,23]. This structure forms small ε-cages and a large 12-ring channel along the c-axis [24,25]. The incorporation of Sr^2+^ ions into this structural framework is feasible [26,27,28,29]. The synthesis of cancrinite is relatively straightforward, employing hydrothermal or high-temperature calcination methods with diverse raw materials, such as silicon and aluminum sources dissolved in NaOH solution, along with specific anions [30,31,32,33]. Coal gangue (CG), a solid waste generated during coal mining, represents a promising resource for utilization [34,35,36]. However, scarce reports exist on utilizing CG as a raw material for solidifying radioactive waste. Notably, the Sr/Al molar ratio in CG is approximately 1, which closely aligns with the Sr/Al molar ratio observed in cancrinite [37,38]. Therefore, CG holds potential as a viable raw material for the synthesis of cancrinite for the purpose of solidifying Sr radionuclides.

This paper reports on the successful preparation of cancrinite for the solidification of Sr^2+^ from Sr nitrate using hydrothermal treatment of CG. The study also investigates the effects of alkali activator concentration, crystallization temperature, and time on cancrinite synthesis, and assesses the maximum Sr capacity and chemical durability of cancrinite synthesized from CG using the PCT method.

## 2. Materials and Methods

### 2.1. Raw Material

**Figure 1:**
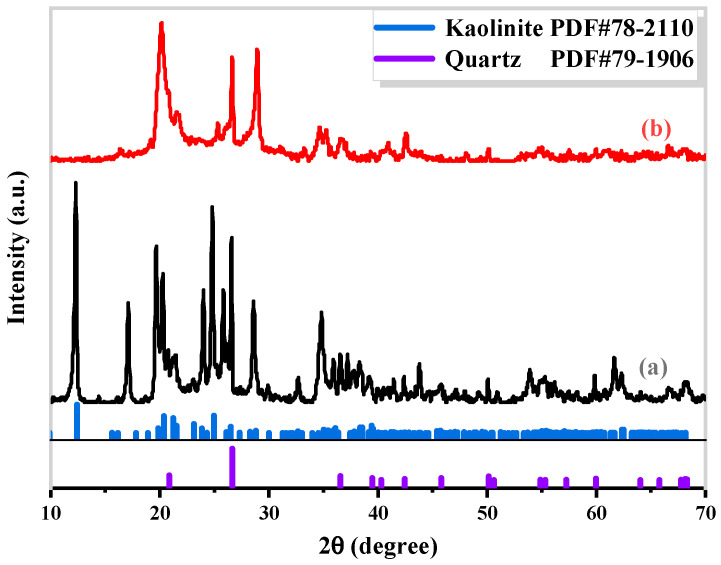
XRD spectra of the coal gangue (**a**) and coal gangue calcined at 900 °C for 5 h (**b**).

The CG samples used in this study were obtained from Shanxi Province. Their chemical and mineral compositions were determined through powder X-ray fluorescence (XRF) and X-ray diffraction (XRD), respectively. The results are presented in Table 1 and Figure 1.

The main chemical components of CG were found to be SiO_2_ (42.93 wt%) and Al_2_O_3_ (30.20 wt%), as expected. After 5 h of decarburization calcination at 900 °C, the contents of SiO_2_ and Al_2_O_3_ in CG increased to 47.15 wt% and 34.58 wt%, respectively. The resulting Al/Si molar ratio was approximately 1, and no additional Si and Al sources were required. XRD analysis of CG before and after calcination (Figure 1) revealed that the dominant minerals in natural CG were kaolinite and quartz. The calcined sample displayed the characteristics of an X-ray amorphous state, except for some platelet diffraction peaks of quartz, with no obvious crystal diffraction peaks. This indicates that the calcination treatment destroyed the initially stable crystal structure of CG, enhancing its activity and contributing to the formation of silicate and aluminate monomers through fusion with alkali. To enhance the purity of cancrinite crystals, calcined CG was employed as the raw material in this experiment.

### 2.2. Experimental Section

#### 2.2.1. Hydrothermal Processing

Calcined CG was combined with Sr(NO_3_)_2_ at specific Sr/Al(Si) molar ratios to serve as the initial materials. This blend was subsequently mixed with NaOH at a predetermined molar ratio. The resulting mixture underwent thorough stirring, facilitated by a magnetic agitator, after the addition of the appropriate quantity of deionized water to achieve uniform pastes. These pastes were molded into cubes (1 × 1 × 1 cm) and left undisturbed at room temperature overnight. Upon demolding, the specimen underwent curing in a Teflon-lined hydrothermal reactor exposed to saturated steam pressure ranging from room temperature to 180 °C for a duration spanning 0 to 24 h. Post-hydrothermal treatment, the synthesized samples were dried at 105 °C for 8 h to facilitate subsequent measurement and characterization.

**Figure 2:**
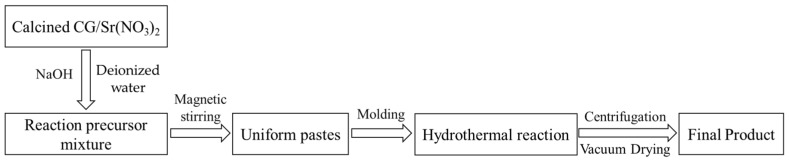
Sr-cancrinite hydrothermal synthesis process flowchart.

The detailed process used for the hydrothermal synthesis of Sr-cancrinite is illustrated in Figure 2.

#### 2.2.2. Single Factor Test

The study systematically explored the effects of NaOH concentration, crystallization temperature, and crystallization time on the immobilization of Sr^2+^. Initially, to assess the influence of NaOH concentration on cancrinite’s crystallinity and purity, experiments were conducted at 180 °C for 24 h with varying relative molar concentrations from 0 to 8 M of NaOH. Subsequently, the impact of the crystallization temperature was examined by varying the temperature from 90 to 180 °C under a fixed 6 M NaOH concentration for 24 h. Following this, the influence of crystallization time on cancrinite synthesis was investigated by setting the time from 0 to 24 h under 6 M NaOH at 180 °C. Finally, the study delved into the effects of Sr/Al(Si) ratios on cancrinite formation, systematically varying the ratios as 0, 1/6, 1/3, 1/2, and 2/3. This comprehensive series of experiments was designed to pinpoint the optimal conditions for the effective immobilization of Sr^2+^.

#### 2.2.3. Sample Characterization and Chemical Durability Testing

The samples underwent characterization of their crystalline phases and morphology using X-ray diffractometry (XRD, EMPYREAN, PANalytical, Almelo, The Netherlands) for phase identification and scanning electron microscopy (SEM, JEOL, JSM-T001F, Tokyo, Japan) for morphology analysis. The chemical stability of Sr-cancrinite was evaluated using the product consistency test (PCT) leaching method [39].

Ion leaching tests were conducted by grinding and filtering the samples through a 100–200 mesh sifter. Subsequently, 1 g of the resulting powder was mixed with 10 mL of deionized water in a Teflon container. The sealed Teflon container was then placed into a stainless vessel and maintained at 363 K for a duration of 168 ± 1 h. The concentration of Sr^2+^ in the leaching solution was analyzed via inductively coupled plasma emission spectrometry (ICP-OES, Agilent 720, Santa Clara, CA, USA). Normalization of the sample concentrations was performed using Equation (1):

(1) $$NC_{i}=\frac{C_{i}}{f_{i}}$$

where *NC_i_* is the normalized concentration of element *i* in the leachate (g·L^−1^), *C_i_* is the concentration of element *i* in the leachate (g·L^−1^), and *f_i_* is the mass fraction of element *i* in the sample.

## 3. Results and Discussion

### 3.1. The Effect of NaOH

**Figure 3:**
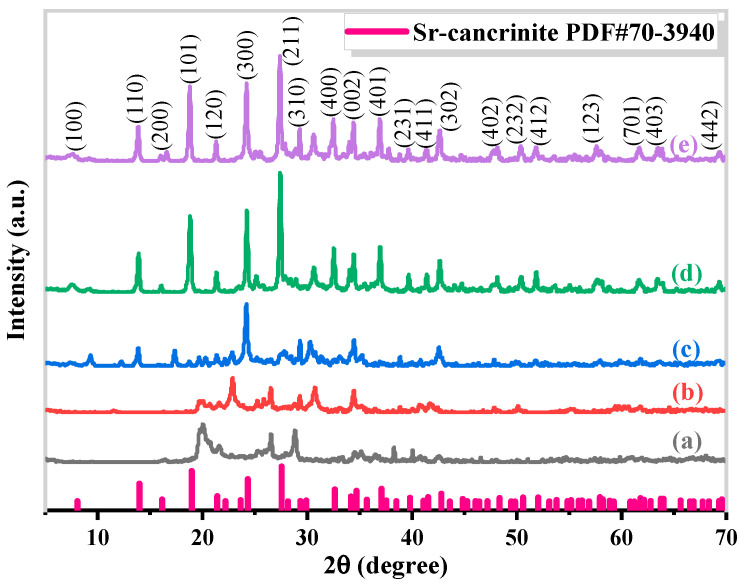
XRD patterns of specimens obtained under different NaOH concentrations when the crystallization temperature and crystallization time were 180 °C and 24 h, respectively (NaOH concentrations: (**a**) 0 M, (**b**) 2 M, (**c**) 4 M, (**d**) 6 M, and (**e**) 8 M).

**Figure 4:**
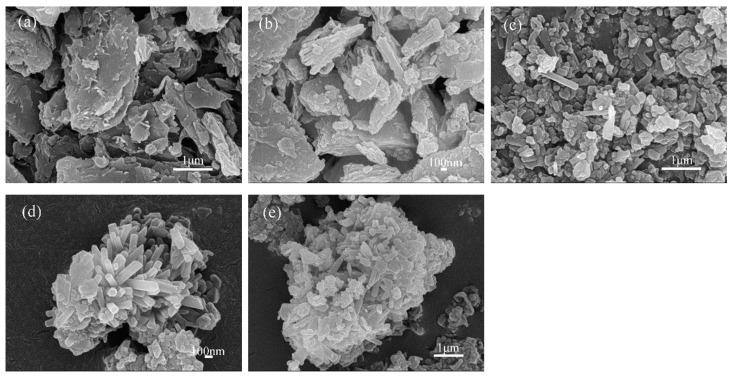
SEM photos of specimens obtained under different NaOH concentrations when the crystallization temperature and crystallization time were 180 °C and 24 h, respectively (NaOH concentrations: (**a**) 0 M, (**b**) 2 M, (**c**) 4 M, (**d**) 6 M, and (**e**) 8 M).

The synthesis of Sr-cancrinite is greatly influenced by the concentration of NaOH due to the framework structure of cancrinite. In order to investigate this effect, the NaOH concentration was varied between 0–8 M while keeping all other conditions constant. Figure 3 presents the XRD patterns of the samples obtained at different NaOH concentrations, while Figure 4 displays the corresponding SEM images.

The XRD patterns in Figure 3a show that the samples existed in an amorphous form similar to the calcined CG before NaOH was added. The SEM images in Figure 4a,b indicate the absence of regular crystals when the NaOH concentration was less than 4 M, and Figure 4c shows only a small amount of columnar cancrinite crystals. However, at a NaOH concentration of 6 M, diffraction peaks of cancrinite crystals were observed, as shown in Figure 3d and reflected in Figure 4d. When the NaOH concentration was 4 M, the grains were less developed, but the number of smooth columnar crystals increased when the NaOH concentration was increased to above 6 M. This observation indicates that cancrinite can be easily synthesized from CG when a sufficient alkali activator is used. The alkali activator induces the dissolution of silica–alumina oxides in CG and enables sufficient synthesis of silica and aluminum tetrahedra to form the silica–alumina cage structure of cancrinite. Furthermore, the ideal chemical formula of Sr-cancrinite [Na_6_Sr(AlSiO_4_)_6_(NO_3_)_2_] indicates coordination numbers of eight for cations and two for anions. In order to satisfy the chemical stability of the Al-Si framework, six cations supplied by NaOH are required to occupy the remaining cation sites, in addition to Sr ions occupying two ionic bonding positions. The synthesized Sr-cancrinite crystals were observed to have a higher particle count and purity when the NaOH concentration was 6 M. Therefore, this concentration was chosen to ensure the reproducibility and consistency of subsequent experiments.

### 3.2. The Effect of Hydrothermal Temperature and Time

**Figure 5:**
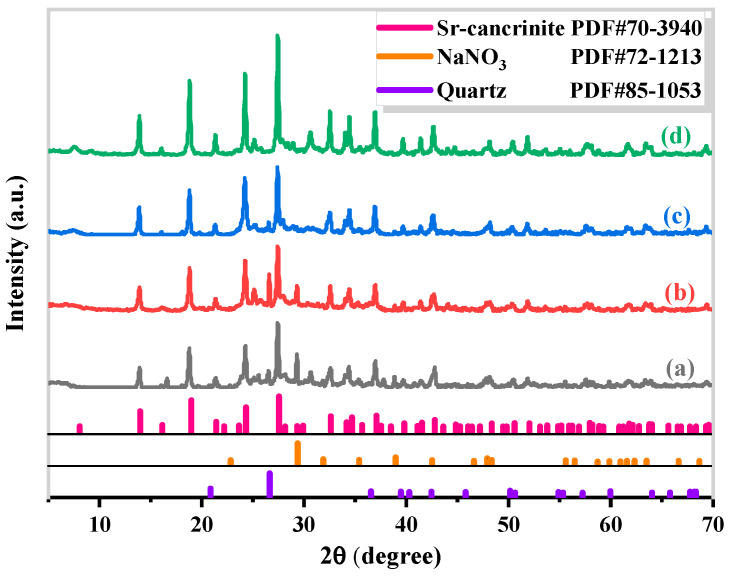
XRD patterns of specimens obtained under different crystallization temperatures when NaOH concentration and crystallization time were 6 M and 24 h, respectively (crystallization temperatures: (**a**) 30 °C, (**b**) 90 °C, (**c**) 120 °C, and (**d**) 180 °C).

**Figure 6:**
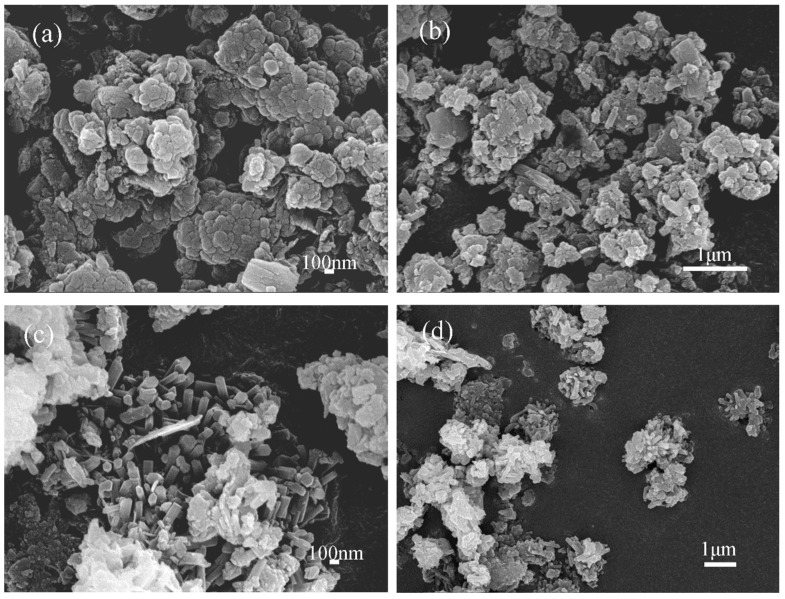
SEM photos of specimens obtained under different crystallization temperatures when NaOH concentration and crystallization time were 6 M and 24 h, respectively (crystallization temperatures: (**a**) 30 °C, (**b**) 90 °C, (**c**) 120 °C, and (**d**) 180 °C).

The formation process of Sr-cancrinite grains is primarily determined by the rate of aluminosilicate gel formation and nucleus growth during hydrothermal treatment, and increasing the hydrothermal temperature can accelerate the synthesis process [40]. Moreover, as hydrothermal time and temperature change, the crystals may undergo phase transformation. To investigate the effects of both the crystallization temperature and time on the synthesis of Sr-cancrinite, the crystallization temperature was raised from room temperature to 180 °C, and the crystallization time was increased from 0 to 24 h while keeping other conditions constant. Figure 5 and Figure 6 display the XRD results and SEM photographs of the Sr-cancrinite samples prepared at different temperatures.

The XRD patterns reveal that the crystalline phase before hydrothermal treatment is primarily Sr-cancrinite with small amounts of quartz and sodium nitrate. The diffraction peaks of quartz and sodium nitrate vanished when the hydrothermal temperature was increased to 120 °C. Moreover, the intensity of the diffraction peaks of Sr-cancrinite increased with the rise in the hydrothermal temperature. By analyzing XRD images, the average grain sizes of cancrinite prepared at room temperature and 180 °C were estimated to be around 384 Å and 460 Å, respectively, indicating that increasing the temperature promoted the growth of grains. Furthermore, SEM images showed that numerous small, spherical crystals existed at room temperature, which gradually grew into short rod-shaped crystals with the increase in temperature and ultimately developed into hexagonal long columnar crystals with characteristic morphology.

**Figure 7:**
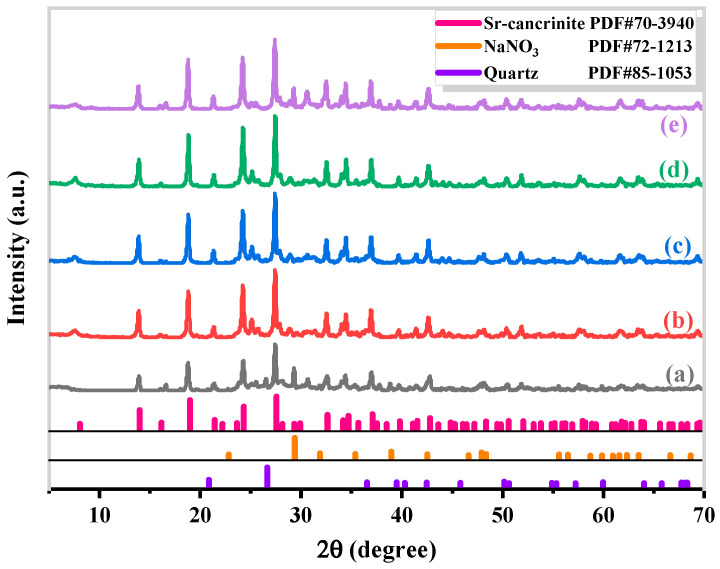
XRD patterns of specimens obtained under different crystallization times when NaOH concentration and crystallization temperature were 6 M and 180 °C, respectively (crystallization times: (**a**) 0 h, (**b**) 3 h, (**c**) 6 h, (**d**) 12 h, and (**e**) 24 h).

**Figure 8:**
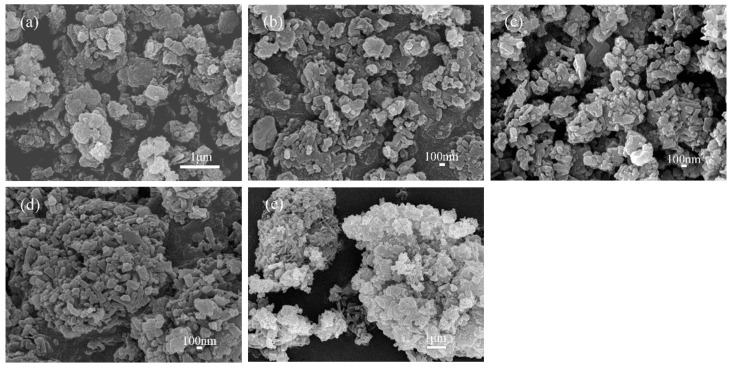
SEM photos of specimens obtained under different crystallization times when NaOH concentration and crystallization temperature were 6 M and 180 °C, respectively (crystallization times: (**a**) 0 h, (**b**) 3 h, (**c**) 6 h, (**d**) 12 h, and (**e**) 24 h).

Figure 7 and Figure 8 present the XRD patterns and SEM images, respectively, of Sr-cancrinite samples prepared at various hydrothermal times.

Before hydrothermal treatment (0 h), the XRD patterns showed the presence of a Sr-cancrinite phase, along with a small amount of quartz and sodium nitrate. After 3 h of hydrothermal treatment, the quartz and sodium nitrate phases disappeared. Additionally, the peak intensity of Sr-cancrinite increased progressively with the increasing curing time. The SEM images depicted the formation of small, spherical grains in the sample without hydrothermal treatment, which subsequently evolved into short, rod-shaped grains with time. Finally, after 24 h of hydrothermal treatment, the grain morphology transformed into smooth, long columnar crystals.

The results of the experimental study indicate that the formation of stable Sr-cancrinite is strongly influenced by the curing temperature and time. Based on these results, the optimal conditions for crystallization were determined to be 180 °C and 24 h. Previous research on hydrothermal treatment with different silica–alumina sources for solidifying inorganic waste materials has shown that an acicular zeolite phase transforms to the sodalite phase, eventually leading to the formation of a stable cancrinite phase as the temperature and time are increased [41]. However, in the current study, stable Sr-cancrinite was already present before hydrothermal treatment, and no transformation of the relevant phases was observed. This can be attributed to two factors. Firstly, the Si/Al molar ratio of the starting material was around 1, similar to the structure of cancrinite. Consequently, stable cancrinite crystal nuclei formed at the initial stage of the reaction, followed by hydrothermal time and temperature, which only promoted the ordered growth of the crystals without leading to further phase transformation. During this process, Sr^2+^ ions entered the lattice structure of cancrinite from the open interlayer domains of zeolite and occupied the lattice cage. The localization and fixation of Sr^2+^ were achieved through various polar interactions, including ion–ion interactions, electric dipole–electric dipole interactions, and electric dipole–ion interactions. Secondly, the cancrinite phase was thermodynamically stable, and even under hydrothermal conditions, the energy generated by temperature and pressure could not overcome the phase transition potential of cancrinite or lead to its transformation into other phases.

### 3.3. Effect of Sr/Al(Si) Molar Ratio

**Figure 9:**
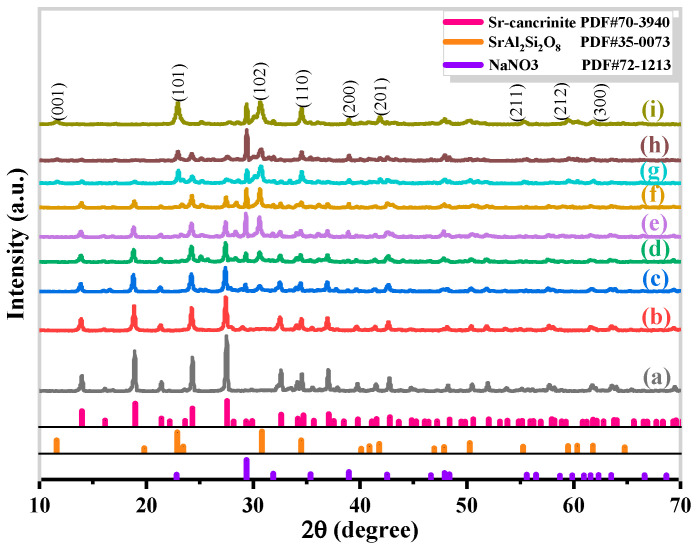
XRD patterns of specimens obtained under different Sr/Al(Si) ratios when NaOH concentration, crystallization temperature, and crystallization time were 6 M, 180 °C, and 24 h, respectively (Sr/Al(Si) ratios: (**a**) 0, (**b**) 1/12, (**c**) 1/6, (**d**) 1/4, (**e**) 1/3, (**f**) 5/12, (**g**) 1/2, (**h**) 7/12, and (**i**) 2/3).

Based on the molecular composition of cancrinite, it can theoretically incorporate four divalent Sr ions into its silica–aluminum framework. To determine the maximum Sr content that can be accommodated, samples were prepared by controlling the Sr/Al(Si) molar ratio in the range of 0–2/3, followed by curing at 180 °C for 24 h. The phase evolution of the samples was investigated using XRD analysis (Figure 9), revealing that a pure and stable Sr-cancrinite phase existed when the Sr/Al(Si) molar ratio was less than 1/6.

**Figure 10:**
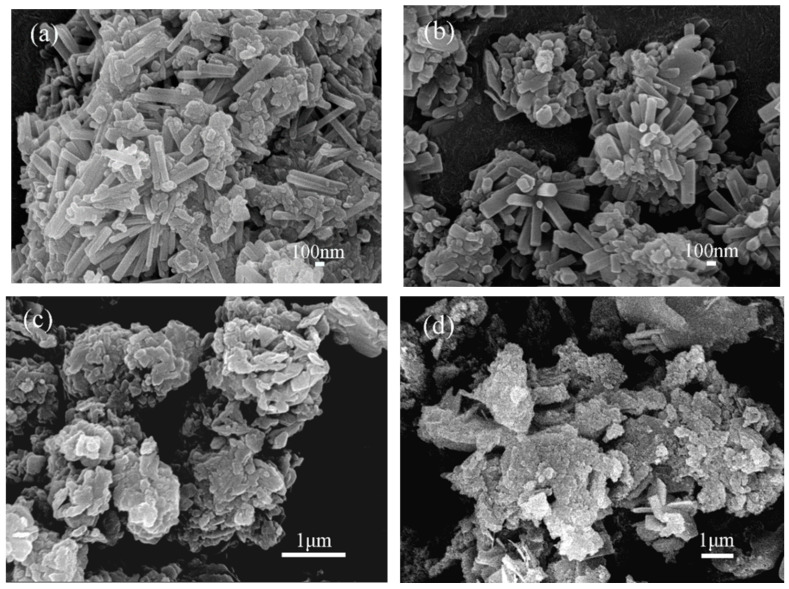
SEM photos of specimens obtained under different Sr/Al(Si) ratios when NaOH concentration, crystallization temperature, and crystallization time were 6 M, 180 °C, and 24 h, respectively (Sr/Al(Si) ratios: (**a**) 1/6, (**b**) 1/3, (**c**) 1/2, and (**d**) 2/3).

However, as the Sr/Al(Si) molar ratio increased, the diffraction peak intensity of the sodium nitrate phase increased, and a new strontium feldspar phase (SrAl_2_Si_2_O_8_) appeared when the ratio reached 1/2. SEM images (Figure 10) confirmed these findings, showing smooth, long columnar cancrinite crystals when Sr/Al(Si) was 1/6 and 1/3, but no representative strontium feldspar crystal structure was observed.

**Figure 11:**
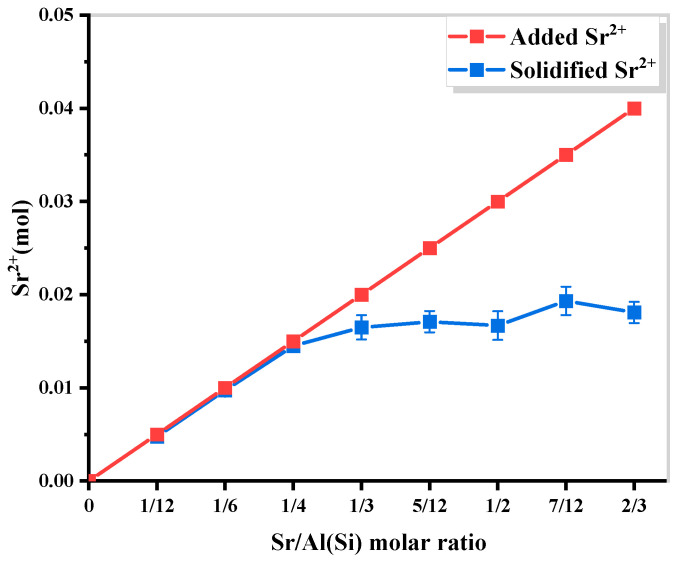
Content of added and solidified Sr^2+^.

The Sr content in the samples was quantified using ICP analysis (Figure 11), which showed that the maximum cured molar fraction of Sr^2+^ was about 1/3 of Al(Si), and the ratio of Sr^2+^ doping to a cured amount decreased significantly when Sr/Al(Si) was greater than 1/6.

The adsorption of Sr^2+^ into the cancrinite crystal structure formed bonds with Al or Si in the lattice, and the bonding strength between Sr and Al or Si was affected by the concentration of Sr^2+^ and the coordination number between Sr^2+^ and Al or Si. Thus, the amount of Sr^2+^ addition and the coordination mode can significantly affect the stability of the cancrinite structure and the interaction of the crystal structure during the curing process.

The experimental results indicate that the quantity of radioactive cations present in cancrinite is significantly influenced by the anion content of the corresponding salt. Specifically, the content of cations should not exceed 25% of the total cation content, with the exception of silica–alumina. The addition of excess strontium salts does not enhance the Sr solidification process, but rather decreases the relative concentration of the alkali activator, resulting in incomplete dissolution of silica–alumina tetrahedra, which are necessary for zeolite formation. Additionally, Sr curing can also occur via the formation of a new strontium feldspar phase (SrAl_2_Si_2_O_8_). However, its curing effect is not as satisfactory as that of cancrinite, and further research is necessary to investigate its content and structural stability. To ensure adequate curing of Sr^2+^ within the crystal structure of cancrinite, the optimal Sr/Al(Si) ratio should be maintained below 1/6.

### 3.4. Sr^2+^ Solidification and Leaching Test

**Figure 12:**
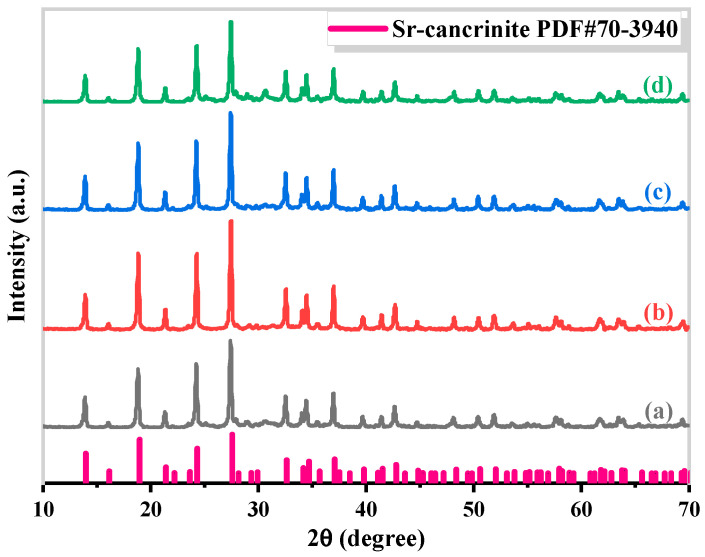
XRD patterns of solidified cancrinite waste specimens (x of Sr_x_Na_8−2x_(AlSiO_4_)_6_(NO_3_)_2_: (**a**) 0.2, (**b**) 0.4, (**c**) 0.6, (**d**) 0.8, and (**e**) 1).

Based on the above experimental results, Sr-cancrinite [Sr_x_Na_8−2x_(AlSiO_4_)_6_(NO_3_)_2_], where x ranges from 0.2 to 1, was successfully synthesized through hydrothermal treatment of CG as the main raw material at 180 °C for 24 h. The immobilization effectiveness of radioactive elements primarily depends on the structure of the main mineral phase of the immobilized material. X-ray diffraction analysis (Figure 12) confirmed that the synthesized sample exhibited a pure and stable cancrinite phase when x < 1.

This finding suggests that Sr-cancrinite can effectively stabilize waste materials under complex natural conditions while preventing the migration of nuclide ions into the environment.

The ion leaching concentrations of Sr-cancrinite, obtained using the PCT method, are presented in Table 2.

The normalized elemental mass release of Sr^2+^ from the samples was consistently low, typically less than that of boroaluminate glasses and calcined ceramics evaluated in other environmental assessments [11]. The solidification effectiveness did not diminish with the increasing Sr^2+^ content. When the Sr^2+^ content was increased to 7.92%, the normalized concentration of leached Sr^2+^ from the cancrinite specimens remained extremely low (6.190 mg/L), and the solidification rate (ratio of solidified Sr content to the total amount) of Sr^2+^ was nearly 100%. These results indicate that cancrinite waste forms possess excellent chemical durability, making them a promising candidate for the immobilization of radioactive elements.

## 4. Conclusions

This paper reports on the successful synthesis of cancrinite crystals capable of immobilizing Sr^2+^ through hydrothermal treatment in an alkaline medium, using CG as the raw material. The study employed XRD, SEM, and PCT leaching tests to evaluate the effectiveness of the synthesized cancrinite crystals in immobilizing Sr^2+^. The findings suggest that stable and efficient immobilization of Sr^2+^ in the crystal structure of the cancrinite can be achieved by controlling the NaOH concentration at 6 M, the hydrothermal temperature at 180 °C, the hydrothermal time at 24 h, and the molar ratio of Sr/Al(Si) at less than 1/6. The resulting cancrinite crystals exhibit low leaching of Sr and demonstrate good thermal and chemical stability.

Compared to traditional curing methods, the hydrothermal treatment method offers several advantages, including simplicity, rapidity, and good feasibility for emergency curing processes in nuclear accidents. The raw material employed is coal gangue, a readily available and cost-effective form of common waste. Furthermore, in comparison to glass and ceramic solidifications, this method exhibits a lower sintering temperature, which is below the boiling point of Sr (approximately 1000 °C) [42]. Consequently, issues such as flux selection and low-temperature densification sintering are alleviated. These findings provide new insights into the potential applications of cancrinite crystals for immobilizing Sr^2+^ and offer a promising approach to mitigate the risks associated with nuclear waste management.

To ensure the viability of this method, the radiation durability of the samples will be tested in subsequent studies. The plan involves simulating the β rays released during the decay of 90Sr and using our accelerator equipment to investigate the sample’s resistance to irradiation. This approach aims to provide a more comprehensive understanding of the sample’s performance under radiation exposure.

## Figures and Tables

**Table 1:** The main chemical composition of coal gangue (wt%).

Ingredients	SiO_2_	Al_2_O_3_	TiO_2_	Fe_2_O_3_	K_2_O	CaO	MgO	Na_2_O	C
Natural CG	42.93	30.20	0.88	0.58	0.43	0.36	0.15	0.10	5.29
Calcined CG	47.15	34.58	1.01	0.73	0.45	0.42	0.17	0.10	1.31

**Table 2:** Ion leaching concentrations of cancrinite according to PCT.

Sample	Mass Ratio of Sr^2+^ (wt%)	Normalized Concentration (mg/L)	Solidification Rate (%)
Na_8_[(SiAl)_6_O_12_](NO_3_)_2_	0	0.000	-
Sr_0.2_Na_7.6_[(SiAl)_6_O_12_](NO_3_)_2_	1.63	2.217	>99.9
Sr_0.4_Na_7.2_[(SiAl)_6_O_12_](NO_3_)_2_	3.24	3.795	>99.9
Sr_0.6_Na_6.8_[(SiAl)_6_O_12_](NO_3_)_2_	4.82	4.561	>99.9
Sr_0.8_Na_6.4_[(SiAl)_6_O_12_](NO_3_)_2_	6.38	5.484	>99.9
SrNa_6_[(SiAl)_6_O_12_](NO_3_)_2_	7.92	6.190	>99.9

## Data Availability

The data are contained within the article.
